# Complete Chloroplast Genomes and Phylogenetic Analysis of Woody Climbing Genus *Phanera* (Leguminosae)

**DOI:** 10.3390/genes15111456

**Published:** 2024-11-12

**Authors:** Yuan Chen, Yanlin Zhao, Wei Wu, Pengwei Li, Jianwu Li, Chang An, Yanfang Zheng, Mingqing Huang, Yanxiang Lin, Quan Yan

**Affiliations:** 1 College of Pharmacy, Fujian University of Traditional Chinese Medicine, Fuzhou 350122, China; 2220408021@fjtcm.edu.cn (Y.C.); 2230408028@fjtcm.edu.cn (Y.Z.); wuweifjwyy@163.com (W.W.); yfzheng@fjtcm.edu.cn (Y.Z.); hmq1115@126.com (M.H.); 2 College of Computer, National University of Defense Technology, Changsha 410073, China; 3 Guangxi Key Laboratory of Plant Conservation and Restoration Ecology in Karst Terrain, Guangxi Institute of Botany, Guangxi Zhuang Autonomous Region and Chinese Academy of Sciences, Guilin 541006, China; pdlc@163.com; 4 Herbarium, Center for Integrative Conservation, Xishuangbanna Tropical Botanical Garden, Chinese Academy of Sciences, Menglun, Mengla 666303, China; ljw@xtbg.org.cn; 5 Fujian Provincial Key Laboratory of Haixia Applied Plant Systems Biology, College of Life Sciences, Fujian Agriculture and Forestry University, Fuzhou 350002, China; ancher0928@163.com

**Keywords:** Leguminosae, Cercidoideae, *Phanera*, chloroplast genome, phylogenetic analysis, evolution

## Abstract

Background: *Phanera* Lour., a genus in the subfamily Cercidoideae of the family Leguminosae, is characterized by woody liana habit, tendrils, and distinctive bilobate or bifoliolate leaves. The genus holds important medicinal value and constitutes a complex group characterized by morphological diversity and unstable taxonomic boundaries. However, limited information on the chloroplast genomes of this genus currently available constrains our understanding of its species diversity. Hence, it is necessary to obtain more chloroplast genome information to uncover the genetic characteristics of this genus. Methods: We collected and assembled the complete chloroplast genomes of nine representative *Phanera* plants, including *Phanera erythropoda*, *Phanera vahlii*, *Phanera aureifolia*, *Phanera bidentata*, *Phanera japonica*, *Phanera saigonensis*, *Phanera championii*, *Phanera yunnanensis*, and *Phanera apertilobata*. We then conducted a comparative analysis of these genomes and constructed phylogenetic trees. Results: These species are each characterized by a typical quadripartite structure. A total of 130–135 genes were annotated, and the GC content ranged from 39.25–42.58%. Codon usage analysis indicated that codons encoding alanine were dominant. We found 82–126 simple sequence repeats, along with 5448 dispersed repeats, mostly in the form of forward repeats. Phylogenetic analysis revealed that 16 *Phanera* species form a well-supported monophyletic group, suggesting a possible monophyletic genus. Furthermore, 10 hypervariable regions were detected for identification and evolutionary studies. Conclusions: We focused on comparing chloroplast genome characteristics among nine *Phanera* species and conducted phylogenetic analyses, laying the foundation for further phylogenetic research and species identification of *Phanera*.

## 1. Introduction

*Phanera* Lour., a member of the subfamily Cercidoideae within the family Leguminosae, constitutes an important part of *Bauhinia* sensu lato species [1]. Predominantly made up of lianas or climbing shrubs (with a minority of trees or shrubs), members of *Phanera* are primarily distributed across tropical and subtropical regions stretching from eastern and southern Asia, with a slight extension into Australia [2,3]. Initially described by Loureiro in 1790, it was based solely on the species *Phanera coccinea* Lour. [4]. The genus has since expanded to include approximately 120–130 species by the time Lewis and Forest recognized its presence in both tropical Asia and the Americas [5].

These climbing plants, including the notable *P. championii* Benth., are esteemed for their medicinal properties [6,7,8,9]. Rich in flavonoids, sterols, aromatic acids, and terpenoids [10,11,12,13], they exhibit a range of pharmacological activities, including but not limited to anti-inflammatory and analgesic properties, treatment of osteoarthritis, inhibition of platelet aggregation, protection against myocardial ischemia, elimination of free radicals, antibacterial effects, as well as neuroprotective effects and anti-tumor effects [7,14,15,16,17]. The medicinal potential of *Phanera* deserves further exploration and utilization.

**Figure 1:**
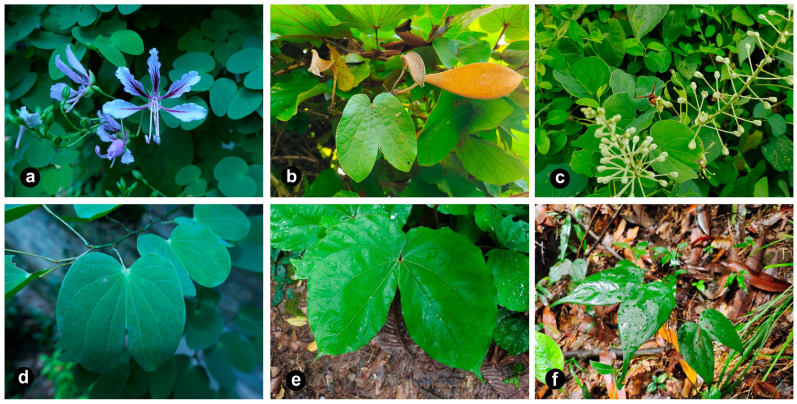
Species morphological diversity within *Phanera*. (**a**) *P. yunnanensis*; (**b**) *P. aureifolia*; (**c**) *P. japonica*; (**d**) *P. saigonensis*; (**e**) *P. erythropoda*; (**f**) *P. championii*.

Taxonomically, *Phanera* presents an intricate group [5]. Apart from *Bauhinia* s.s., there are seven additional genera that share a close affinity with *Phanera*, including *Gigasiphon* Drake, *Tylosema* (Schweinf.) Torre and Hillc., *Barklya* F. Muell., *Cheniella* R. Clark and Mackinder, *Lasiobema* (Korth.) Miq., *Lysiphyllum* (Benth.) de Wit, and *Schnella* Raddi. Species within *Phanera* display significant morphological and molecular heterogeneity (Figure 1) [18]. And the unstable boundaries of the *Phanera* genus have led to several revisions recently concerning its relationship with *Schnella*, *Lasiobema*, and *Cheniella* (Table 1) [3,4,19,20,21,22,23,24,25]. Drawing upon analyses of molecular phylogeny and variations in morphology, Wunderlin reassigned the American clade of *Phanera* to the resurrected *Schnella* Raddi [22,23]. The Legume Phylogeny Working Group (LPWG) treated *Lasiobema* as a synonym of *Phanera*, which is further supported by Sinou [3,26]. In addition, 10 species from the *Phanera* subsect. Corymbosae de Wit were categorized and reassigned under a novel genus, *Cheniella* R. Clark and Mackinder [24,25]. Subsequent research has since augmented the species count within this genus to 16 species and 3 subspecies [27]. To date, the most widely accepted morphological characteristics that distinguish *Phanera* at the generic level from other genera within *Bauhinia* s.l. include its lianescent habit (tendril), 2–3 fertile stamens, a lobed or truncated calyx, and a non-elongated hypanthia [2].

Compared to nuclear genomes, chloroplast (cp) genomes are characterized by their small size, monolepsis, low nucleotide substitution rates, haploidy, and highly conserved genome structure [28]. Due to these attributes, cp genomes are regarded as ideal models for studying biodiversity and evolution and have been successfully employed as super barcodes for molecular marker development and phylogeographic analysis [26,29]. In taxonomic groups with complex traits and challenging morphological identification, the development of molecular markers is particularly important [30]. Due to their difficult-to-distinguish morphological features, taxa like *Phanera* particularly benefit from the development of molecular markers for accurate identification. However, the phylogenetic relationships within *Phanera* based on core DNA barcodes remain uncertain [3]. There is an urgent need to develop molecular markers for *Phanera*, both from the perspective of phylogenetic research and species identification.

Despite the numerous reports on molecular loci of species belonging to Cercidoideae, investigations into the cp genomes of *Phanera* plants are still scant, and no specific comprehensive comparison of complete cp genomes or phylogenetic analysis has been conducted for this genus, impeding our comprehension of its phylogenetic relationships and species diversity. Therefore, this study collected nine representative species of *Phanera* from various regions, including *P. vahlii* (representing India), *P. erythropoda* (southwestern China), *P. championii* (southern China), *P. apertilobata* (southeastern China), *P. japonica* (eastern China and Japan), *P. aureifolia* (Thailand), *P. bidentata* (Malaysia), as well as *P. yunnanensis* (distributed in central and southern China, Thailand, and Myanmar), and *P. saigonensis* (distributed in Cambodia, Laos, Thailand, and Vietnam). The objective was to sequence, assemble, and analyze the cp genomes of these species through high-throughput sequencing technology, thereby uncovering their genetic characteristics at the genetic level. Our primary goal is to investigate the structural variability in the cp genomes of the sampled *Phanera* species and to ascertain their phylogenetic positions based on these nine cp genomes from *Phanera*.

## 2. Materials and Methods

### 2.1. Materials and Extraction of Genomic DNA

We have collected nine representative species from different regions belonging to the *Phanera* genus, which comprises a total of approximately 74 species. These samples were obtained from Yunnan, Hainan, and Fujian in China, and their voucher specimens have been archived in the Herbarium of Fujian University of Traditional Chinese Medicine. Immediately upon collecting these tender leaves, they were preserved in liquid nitrogen for freezing and transported to the lab, where they were kept at −80 °C in preparation for DNA extraction. The extraction of total genomic DNA from the young leaves was carried out using the DNA Quick Plant System (TIANGEN BIOTECH Co., Ltd., Beijing, China).

### 2.2. DNA Sequencing, Assembly, and Annotation

Paired-end libraries of 350 base pairs (bp) were constructed according to the manufacturer’s protocol for genomic DNA library preparation, followed by further quality control measures. We outsourced sequencing to Novogene Bioinformatics Technology Co., Ltd. (Tianjin, China). Utilizing the Illumina NovoSeq 6000 sequencing platform (Illumina, San Diego, CA, USA), we generated 150 bp paired-end reads. Fastqc v0.11.9 (http://www.bioinformatics.babraham.ac.uk/projects/fastqc/, accessed on 20 May 2024) was employed to evaluate the sequencing quality of all raw data, while Fastp v0.23.4 software [31] was then utilized to filter out low-quality reads, trim sequences with suboptimal lengths, and remove adapter sequences from both ends to obtain a set of high-quality clean data.

Thereafter, Getorganelle v1.7.7.0 software [32] was used to assemble the complete cp circular genomes. The annotation process of these genomes was conducted via the Geseq v2.03 [33] and the CPGAVAS2 [34] tools, selecting the congeneric species *P. venustula* (OQ701684) [25] as the reference genome. Redundant annotation information was removed, and abnormal annotation features were manually reviewed and corrected. Additionally, tRNAscan-SE v2.0 software [35] was utilized to verify the tRNA annotations, leading to the final acquisition of annotated information. The cp genomes were visualized through the CPGView website [36]. Both the cp genome sequences and their final annotations for the *Phanera* species have been submitted to GenBank (Table S1).

### 2.3. Codon Usage Bias Analysis

Relative synonymous codon usage (RSCU) represents the relative probability of a specific codon among its synonymous counterparts that encode the same amino acid [37]. It is calculated as the ratio of (the usage frequency of a specific codon) to (the average frequency of all synonymous codons that encode the identical amino acid) [38]. Variations in RSCU values have been observed across diverse species, with research indicating a potential correlation between this metric and the level of gene expression [39]. In this study, CodonW v1.4.2 software [40] was employed to compute and analyze the RSCU of 139 coding sequences (CDS) from 16 cp genomes of *Phanera* (Table S1). The result was plotted using ChiPlot (https://www.chiplot.online/, accessed on 1 October 2024). RSCU exceeding 1 indicates a higher usage frequency for a particular, suggesting a codon preference. Conversely, values below 1 signify a relatively lower usage frequency, while an RSCU of exactly 1 implies a neutral codon preference that is devoid of any significant bias.

### 2.4. Repeat Sequences Analysis

Repeat sequences influence the evolution, genetics, and variation of organisms while also playing an important role in gene expression, transcriptional regulation, and physiological metabolism. Here, the MISA v2.1 tool [41] was used to detect simple sequence repeats (SSRs) in the cp genomes of nine *Phanera* species, with the minimum thresholds for mononucleotide to hexanucleotide repeats set at 10, 5, 4, 3, 3, 3, respectively. In the meantime, the REPuter database (https://bibiserv.cebitec.uni-bielefeld.de/reputer, accessed on 1 October 2024) [42] was used to analyze dispersed repeats within these cp genomes, including four distinct categories: forward (F), reverse (R), palindromic (P), and complementary (C) repeats, with the analysis parameters set to a hamming distance threshold of 3, a cap on the maximum number of computed repeats at 5000, and a minimum repeat size of 30.

### 2.5. Chloroplast Genomes Comparison

The boundary variations in the quadripartite structure contribute to the alterations observed in plant cp genomes. Here, the cp genomes of nine *Phanera* species were examined. Utilizing the CPJSdraw v1.0.0 software [43], the boundaries of the large single copy (LSC), small single copy (SSC), and inverted repeats (IRs) across these genomes were analyzed and compared. Utilizing the mVISTA online tool (https://genome.lbl.gov/vista/mvista/submit.shtml, accessed on 4 October 2024) [44] with *P. erythropoda* as the reference genome, we compared the similarity among the cp genomes of these plants, selecting the Shuffle-LAGAN program. We also extracted both the CDS and intergenic spacer (IGS) sequences of the cp genomes from 16 *Phanera* species (Table S1) for DNA sequence polymorphisms analysis using DnaSP v5.10.01 software [45].

### 2.6. Phylogenetic Tree Construction

We constructed phylogenetic trees using the complete cp genomes of nine *Phanera* plants we assembled here, as well as 25 closely related Cercidoideae species from the NCBI database (https://www.ncbi.nlm.nih.gov/, accessed on 4 October 2024) (Table S1). The outgroup was the most basal species of *Cercis* L. within Cercidoideae, specifically *Cercis chinensis* (MZ128523) [46] and *Cercis canadensis* (KF856619) [47]. The cp genomes mentioned above were aligned using the MAFFT v7.520 software [48], followed by filtering of the aligned sequences with R package alignmentFilter v1.0.0 [49]. Subsequently, phylogenetic trees were constructed employing Maximum Likelihood (ML) and Bayesian Inference (BI) methods. For the ML tree construction, IQ-Tree v2.0.3 software [50] was used to obtain the optimal tree-building model, which was identified as GTR + F + I + G4. Following this, the ML tree was generated through this software, applying a bootstrap value of 1000. When constructing the BI tree with MrBayes v3.2.7a software [51], the optimal model detected by IQ-Tree v2.0.3 software was selected. The MCMC algorithm was executed over a total of 1,000,000 generations, and samples were collected at an interval of every 100 generations. To ensure the accuracy of the results, a burn-in of 25% was applied to discard initial trees, ultimately yielding a tree annotated with posterior probabilities.

## 3. Results

### 3.1. Chloroplast Genomes Features

**Figure 2:**
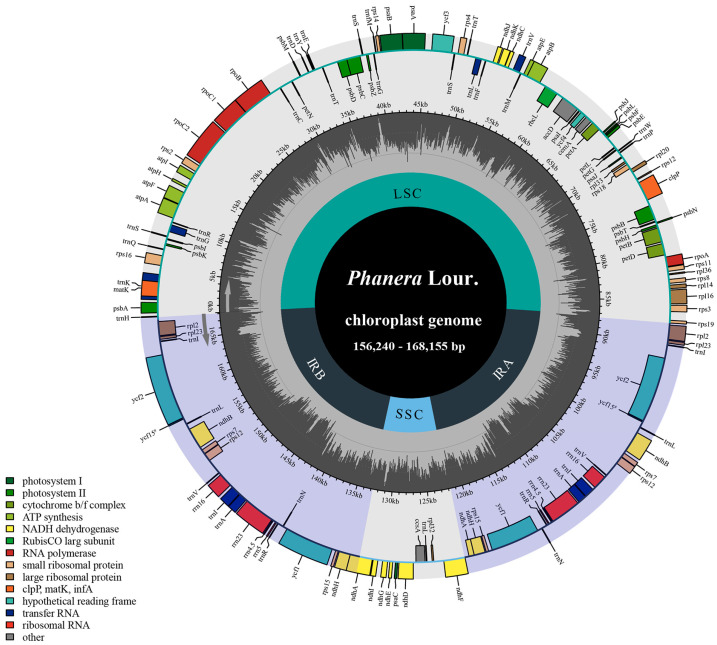
Physical map of the cp genomes in nine *Phanera* species. The gray column chart in the inner circle represents the CG content. The inner and outer sides of the outer circle represent the genes in clockwise and counterclockwise directions, respectively. Different color blocks represent gene groups with different functions, and the functions corresponding to the colors are annotated in the lower left corner.

The complete cp genomes from the nine *Phanera* plants we assembled here were all typical quadripartite structures. We have observed that the total length of these genomes ranged from 156,240 bp (*P. erythropoda*) to 168,155 bp (*P. bidentata*) (Figure 2). Overall, aside from *P. bidentata* having a much longer length compared to other species, the sequence lengths of cp genomes did not exhibit significant variations among different species. LSC region (86,829–89,725 bp) and SSC region (11,035–19,010 bp) were separated by two inverted repeat (IR) regions (25,647–34,934 bp). The nucleotide composition analysis showed that the percentage of guanine-cytosine (GC) varied between 35.91% and 36.36% overall, and the adenine-thymine (AT) content ranged from 63.64% to 64.09%. The distribution of GC and AT contents across four regions was uneven. Among them, the IR regions had the highest GC content (39.25–42.58%), followed by LSC (33.92–34.24%), and then the SSC regions (29.76–30.96%). Similarly, the AT content varied among the four regions and was higher than the GC content in all four regions.

Based on the complete gene annotation, the cp genomes of nine *Phanera* species contained from 130 to 135 genes. Specifically, 85–90 were annotated as protein-coding genes, 36–37 as tRNA genes, and eight as rRNA genes (Table S2). Of these protein-coding genes, 46 are related to photosynthesis, 31 genes are associated with self-replication, and there are six other genes, as well as six genes with unknown functions. Among the 37 tRNA genes, seven genes had two copies: *trnA-UGC*, *trnI-CAU*, *trnI-GAU*, *trnL-CAA*, *trnN-GUU*, *trnR-ACG*, *trnV-GAC*. And all eight rRNA genes (*rrn4.5*, *rrn5*, *rrn16*, and *rrn23*) also existed in two copies. Moreover, among these 135 genes, 20 genes contained one intron comprising 10 protein-coding genes (*atpF*, *petB*, *petD*, *ndhA*, *ndhB*, *rpoC1*, *rpl2*, *rpl16*, *rps16* and *rps18*) and six tRNA genes (*trnA-UGC*, *trnG-UCC*, *trnI-GAU*, *trnK-UUU*, *trnL-UAA*, and *trnV-UAC*), while four genes (*accD*, *rps12*, *ycf3*, *clpP*) contained two introns.

### 3.2. Relative Synonymous Codon Usage Analysis

**Figure 3:**
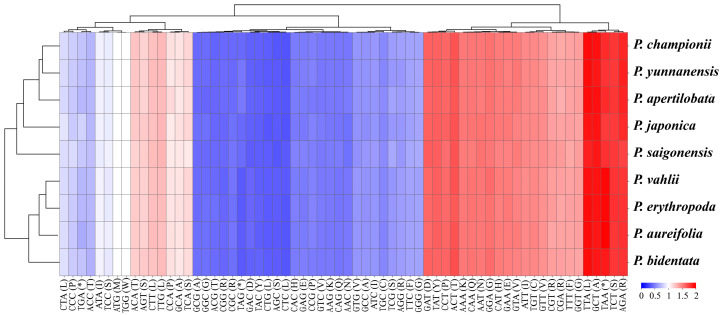
The RSCU values of each amino acid in the chloroplast genomes of nine *Phanera* species.

To elucidate the codon usage patterns in the cp genome of nine *Phanera* species, we calculated RSCU values across its protein-coding gene sequences. Among 64 codons in *Phanera* species, including two codons with RSCU = 1 (AUG, UGG) and three termination codons (UAA, UAG, UGA), the usage frequency spans from 18,860–20,640 (Figure 3, Tables S3–S11). Excluding three termination codes, all codons encoding amino acids, those with high frequencies (RSCU > 1.6) included UUA for leucine, GCU for alanine, AGA for arginine, UCU for serine, ACU for threonine, UAU for tyrosine and GAU for aspartic acid. The codon with the highest frequency was AUU, with counts of 797–889, accounting for 4.23–4.30%. It encoded the amino acid isoleucine (Ile) with a relative synonymous codon usage (RSCU) of 1.46–1.48. The codon UGC, reaching a total count of 55–61, had the lowest frequency, accounting for 0.27–0.30%. It encoded the amino acid cysteine (Cys) with an RSCU of 0.51–0.54. There were 29 RSCU > 1, of which 28 amino acids ended in A/U (96.55%) and one terminated in G/C (3.45%). There were 30 RSCU < 1, 28 of which ended in G/C (93.33%), and only two ended in A/U (6.67%). In summary, the majority of codons with RSCU > 1 ended in A/U, indicating that these are the preferred codons of the *Phanera* species cp genomes. Conversely, among the codons where RSCU < 1, most ended in G/C, suggesting that these are the non-preferred codons of *Phanera* species. However, the two codons with RSCU = 1 showed no preference and encoded methionine and tryptophan acid separately.

### 3.3. Dispersed Repeats and SSRs Analysis

**Figure 4:**
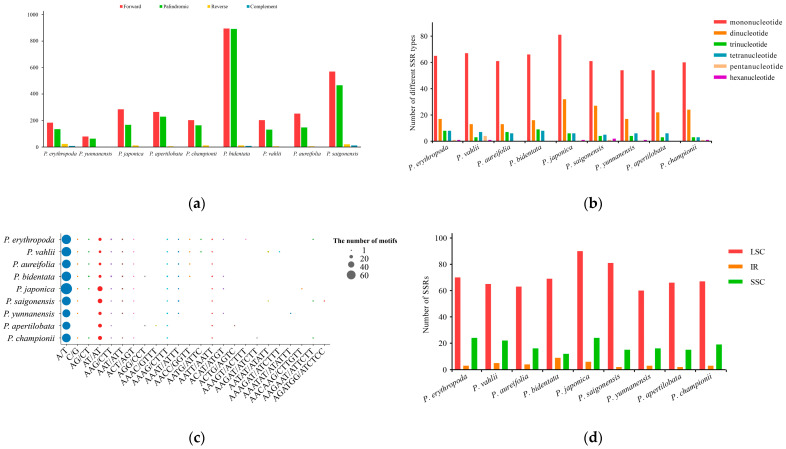
Identification of dispersed repeat sequences and SSRs in the cp genomes of nine *Phanera* species. (**a**) the number of dispersed repeat sequences of varying lengths within the cp genomes of these species; (**b**) the number of SSRs in the cp genomes of these species; (**c**) the number of SSRs with different motifs; (**d**) The number of SSRs in LSC, SSC, and IRs of the cp genomes of these species.

Utilizing REPuter (https://bibiserv.cebitec.uni-bielefeld.de/reputer, accessed on 1 October 2024), repeat sequences exceeding 30 bp in length within the cp genomes were identified. A total of 5448 dispersed repeat sequences were detected, categorized into 79–895 forward repeats, 64–891 palindromic repeats, 1–23 reverse repeats, and 0–11 complementary repeats (Figure 4a). The majority of these sequences ranged between 30–39 bp (55.38%), followed by 40–49 bp (18.96%), and the least frequent were those spanning 70–79 bp (2.85%) (Figure S1). Significantly, in *P. bidentata*, we detected the highest number of repetitive sequences, with more sequences within the range of 30–39 bp than other species. These repetitive sequences contributed to *P. bidentata* having the largest cp genome among these species. The IR region contained 81.94% of all repetitive sequences, making it the primary region of distribution for such sequences. Among them, *P. bidentata* had the highest proportion of repetitive sequences in the IR region (1746), accounting for 41.03%, while the repetitive types of *P. bidentata* located in the SSC region (3) accounted for 1.75%, which was relatively less compared to other species (Figure S2). In addition, the proportions of duplication in the LSC and SSC regions were 3.10% and 0.17% of all regions, respectively.

The SSR analysis revealed that the cp genomes of nine *Phanera* species contained 82–126 SSRs, including 54–81 mononucleotide repeats (63.53–64.29%), 13–32 dinucleotide repeats (13.68–25.40%), 3–9 trinucleotide repeats (3.16–7.14%), 3–8 tetranucleotide repeats (3.26–6.35%), 0–4 pentanucleotide repeats (0–3.17%), and 0–2 hexanucleotide repeats (0–1.59%) (Figure 4b). *P. aureifolia*, *P. apertilobata*, and *P. bidentata* failed to detect pentanucleotide and hexanucleotide repeats, while *P. yunnanensis* and *P. japonica* did not detect pentanucleotide sequences. Among all *Phanera* species, *P. japonica* had higher mononucleotide repeats than other species. In addition, the proportions of mononucleotide repeat A/T and C/G types were 62.70% and 0.79%, respectively (Figure 4c), similar to that of most angiosperms, indicating that A/T type mononucleotide repeats may be the most abundant repeat type in SSRs. The higher number of A/T repeats in *P. japonica* compared to other species indicated diversities among different *Phanera* species. In addition, the number of dinucleotide AT/AT was second only to A/T, which further confirmed the activity of A/T bases in the cp genomes. Furthermore, *P. erythropoda*, *P. vahlii*, *P. aureifolia*, *P. yunnanensis,* and *P. bidentata* had similar AT/TA type proportions, supporting their genetic relationship. There were 60–90 SSRs distributed in LSC (accounting for 67.01–81.00% of all regions within the cp genome) (Figure 4d). Among them, the LSC of *P. saigonensis* exhibited the largest proportion of SSRs, while the LSC of *P. japonica* contained the highest count of SSRs, totaling 90 and accounting for 71.43% of all regions within its cp genome. And the SSRs were dispersed across SSC regions, with a count ranging from 12 to 24 SSRs, accounting for 12.12–24.00%. In addition, the IGS region exhibits the highest frequency of SSR distributions, with a range of 58–93 SSRs (58.59%~76.09%), followed closely by the CDS region with 8–24 SSRs (8.00%~24.24%), and the intron region with 8–17 SSRs (9.41–17.17%) (Figure S3). *P. saigonensis* in CDS and *P. apertilobata* in intron had far fewer SSRs (eight each) compared to other species.

### 3.4. Boundaries of Junction Sites Analysis

**Figure 5:**
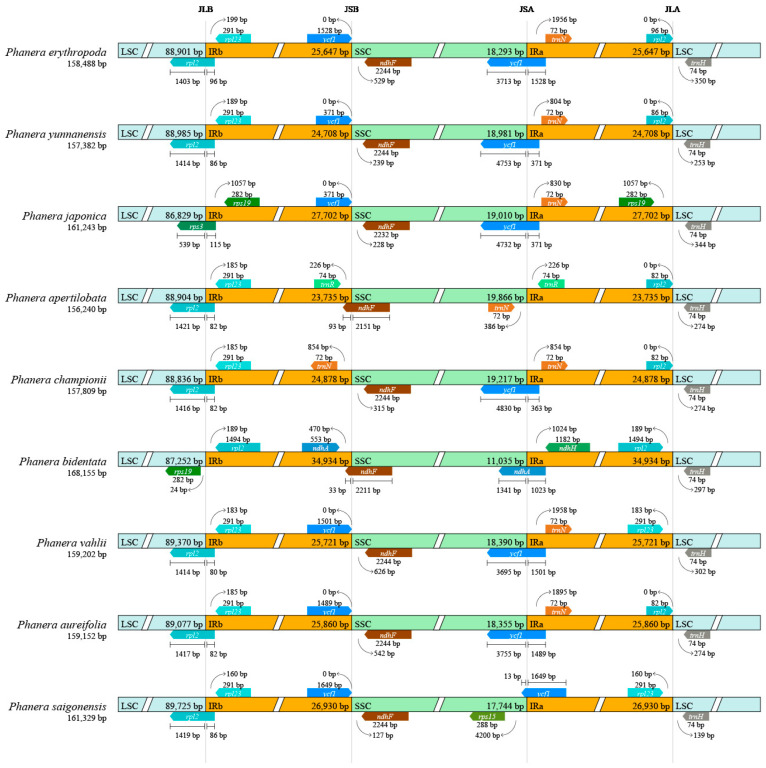
Comparison of LSC, SSC, and IRs boundaries in the cp genome of nine *Phanera* species.

A comparative analysis of four boundaries across the cp genomes of nine *Phanera* species revealed relative conservation of the cp genome structure despite some variation in sequence length and dynamic processes of contraction and expansion observed at the boundaries of their various partitions (Figure 5).

At the boundary between LSC and IRb, the junction points for *P. erythropoda*, *P. yunnanensis*, *P. apertilobata*, *P. championii*, *P. vahlii*, *P. aureifolia*, and *P. saigonensis* were all located within the coding region of the *rpl2* genes. The *rpl2* gene of *P. bidentata* was positioned 189 bp upstream of IRb. The *rpl23* genes of *P. erythropoda*, *P. yunnanensis*, *P. apertilobata*, *P. championii*, *P. vahlii*, *P. aureifolia*, and *P. saigonensis* were located 199 bp, 189 bp, 185 bp, 185 bp, 183 bp, 185 bp, and 160 bp upstream of the LSC, respectively. Notably, in *P. japonica*, the boundary between LSC and IRb was within the coding region of the *rps3* gene. The *rps19* gene was only found at the LSC and IRb boundary in *P. bidentata* and *P. japonica*, but its position and distance vary. In *P. bidentata*, the *rps19* gene was located 24 bp downstream of LSC, while in *P. japonica*, it was positioned 1057 bp upstream of IRb.

Except for *P. bidentata*, *P. championii*, and *P. apertilobata*, the IRb and SSC boundary genes of these *Phanera* species showed conservation. Downstream of IRb, the boundary gene of *P. apertilobata* was *trnR*, while for *P. championii* and *P. bidentata*, it was *trnN* and *ndhA*, respectively. For all other species, the gene at this boundary was *ycf1*. The IRb and SSC boundaries of *P. bidentata* and *P. apertilobata* were both located within the coding region of the *ndhF* gene. In contrast, the *ndhF* genes of *P. erythropoda*, *P. yunnanensis*, *P. championii*, *P. vahlii*, *P. aureifolia*, *P. japonica*, and *P. saigonensis* were positioned 529 bp, 239 bp, 228 bp, 315 bp, 626 bp, 542 bp, 228 bp, and 127 bp upstream of the SSC, respectively.

Considerable variations were observed in the cp genome of these species at the SSC and IRa boundaries. Except for *P. apertilobata*, the boundaries of the remaining species were located within *ycf1* genes. For most species, the genes at the SSC and IRa boundaries were *ycf1*, and *trnN*, with the *trnN* genes of *P. erythropoda*, *P. yunnanensis*, *P. japonica*, *P. championii*, *P. vahlii*, and *P. aureifolia* positioned 1956 bp, 804 bp, 830 bp, 854 bp, 1958 bp, and 1895 bp upstream of IRa, respectively. In contrast, the mutations of *P. apertilobata* and *P. bidentata* were larger than those of other species. *P. apertilobata* had both *trnN* and *trnR* genes at this boundary, while *P. bidentata* had the genes *ndhA* and *ndhH*.

The boundaries between IRa and LSC also demonstrated conservation, with each species possessing *trnH* gene located 139–350 bp upstream of the LSC. In *P. bidentata*, the *rpl2* gene was situated 189 bp downstream of IRa. The *rpl2* gene in *P. erythropoda* was at the downstream boundary of IRa, whereas in other species, the *rpl2* genes at this boundary were pseudogenized. Notably, only *P. japonica* had the *rps19* gene at the downstream boundary of IRa.

### 3.5. Sequence Variation Analysis

**Figure 6:**
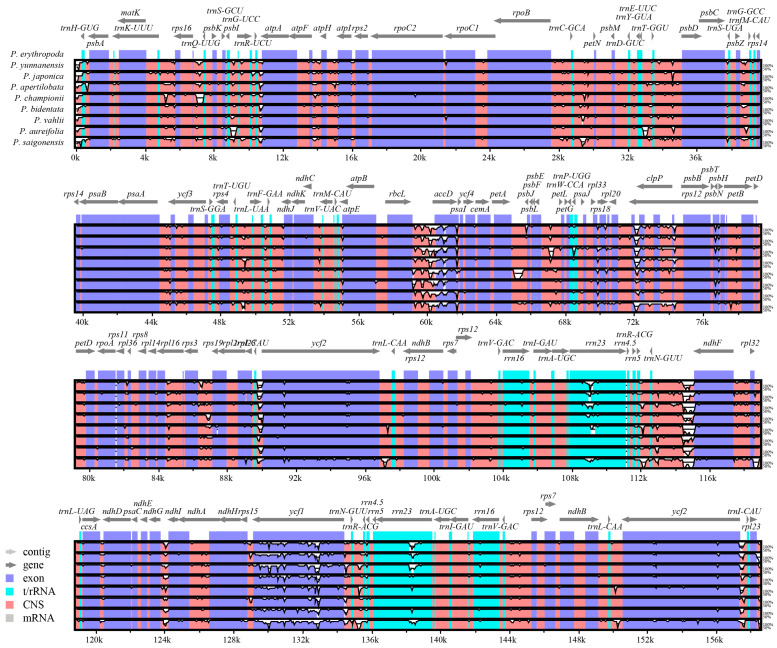
Sequence similarity of the cp genomes between nine *Phanera* species was analyzed using mVISTA with *P. erythropoda* as a reference. The horizontal axis represents the gene position in the reference genome of *P. erythropoda*, while the vertical axis indicates the similarity ranging from 50% to 100%. Exons, t/rRNAs, and conserved noncoding sequences (CNS) are represented by different colors in the lower left corner.

Using the cp genome of *P. erythropoda* as a reference, we employed the mVISTA online tool (https://genome.lbl.gov/vista/mvista/submit.shtml, accessed on 4 October 2024) [44] to compare this sequence with the cp genomes of *P. yunnanensis*, *P. japonica*, *P. apertilobata*, *P. championii*, *P. vahlii*, *P. aureifolia*, *P. bidentata*, and *P. saigonensis* (Figure 6). Among these genomes, non-coding regions exhibited high variability, whereas the coding regions displayed relatively low variation. Notably, the variations were predominantly observed in IGS regions of adjacent genes. The SSC region of the cp genomes showed less variation compared to the LSC and IR regions, with the LSC region displaying the highest degree of variation.

**Figure 7:**
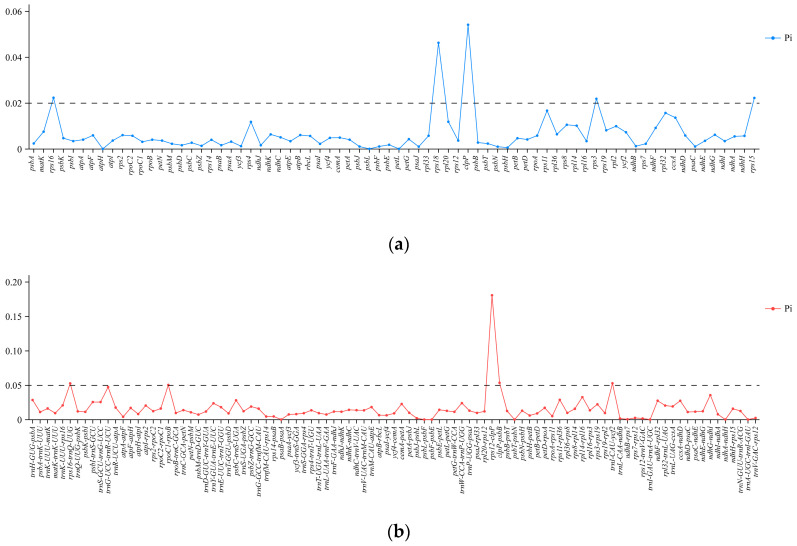
Nucleotide polymorphism (Pi) in the cp genomes of 16 *Phanera* species. (**a**) Pi of the common CDS regions in the cp genomes. (**b**) Pi of the common IGS regions.

Upon extracting 74 CDS and 97 IGS sequences from the cp genomes of 16 species, the nucleotide polymorphism (Pi) values were calculated. The nucleic acid variation analyses showed that IGS had more polymorphisms than the CDS regions. The hypervariable regions comprised the regions of CDS: *clpP* (0.05411), *rps18* (0.04625), *rps16* (0.02229), *rps15* (0.02219)*, rps3* (0.02184) (Pi > 0.02) (Figure 7a). Among the five hypervariable regions, *rps16*, *rps3*, *rps18*, and *clpP* were located in the LSC, and *rps15* was located in the SSC.

We have identified the highly variable IGS regions as follows: *rps12*-*clpP* (0.18062), *clpP*-*psbB* (0.05340), *trnI-CAU*-*ycf2* (0.05269), *rps16*-*trnQ-UUG* (0.05255), *rpoC1*-*rpoB* (0.05031) (Pi > 0.05) (Figure 7b). Among the four hypervariable IGS regions, *rps16*-*trnQ-UUG*, *rpoC1*-*rpoB*, *rps12*-*clpP*, and *clpP*-*psbB* were located in LSC. One region, *trnI-CAU-ycf2*, was located in IRs.

### 3.6. Phylogenetic Analysis

**Figure 8:**
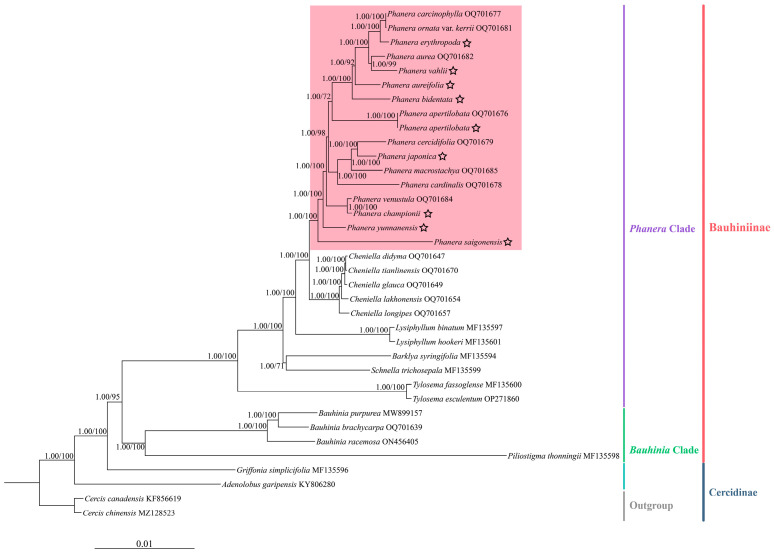
The phylogenetic relationships of the subfamily Cericidoideae were inferred using the complete cp genomes based on BI and ML methods. The support values on the branches are presented in the order of PP_BI_/BS_ML_ (with 1.00/100 support values). The pink box indicates species belonging to *Phanera.* The lineages of these species are labeled on the right. Outgroups are *C. chinensis* (MZ128523) and *C. canadensis* (KF856619). The Cercidoideae species used to construct this phylogenetic tree include *Adenolobus garipensis* (KY806280), *Barklya syringifolia* (MF135594), *Bauhinia brachycarpa* (OQ701639), *Bauhinia purpurea* (MW899157), *Bauhinia racemosa* (ON456405), *Cheniella didyma* (OQ701647), *Cheniella glauca* (OQ701649), *Cheniella lakhonensis* (OQ701654), *Cheniella longipes* (OQ701657), *Cheniella tianlinensis* (OQ701670), *Griffonia simplicifolia* (MF135596), *Lysiphyllum binatum* (MF135597), *Lysiphyllum hookeri* (MF135601), *P. apertilobata* (OQ701676), *Phanera aurea* (OQ701682), *Phanera carcinophylla* (OQ701677), *Phanera cardinalis* (OQ701678), *Phanera cercidifolia* (OQ701679), *Phanera macrostachya* (OQ701685), *Phanera ornata* var. *kerrii* (OQ701681), *Phanera venustula* (OQ701684), *Piliostigma thonningii* (MF135598), *Schnella trichosepala* (MF135599), *Tylosema esculentum* (OP271860), *Tylosema fassoglense* (MF135600), as well as nine *Phanera* species assembled in this study. The cp genomes from nine *Phanera* plants assembled here are marked with a star.

Phylogenetic trees were reconstructed using nine *Phanera* cp genomes obtained from sequencing and 27 other species of Cercidoideae from NCBI to determine the phylogenetic relationship of *Phanera* species and the genetic relationships of *P. erythropoda*, *P. vahlii*, *P. aureifolia*, *P. bidentata*, *P. yunnanensis*, *P. japonica*, *P. apertilobata*, *P. championii*, and *P. saigonensis* (Figure 8). The phylogenetic trees based on ML and BI methods demonstrated an overall high level of support, with high resolution at the trunk. The phylogenetic trees showed that the species of the subfamily Cercidoideae are divided into two major subtribes, Cercidinae and Bauhiniinae. Cercidinae included species from *Cercis* L. *Adenolobus* (Harv. ex Benth. and Hook.f.) Torre. and Hillc., and *Griffonia* Baill., and was located at the base of the subfamily Cercidoideae. Bauhiniinae was divided into two major branches, including *Bauhinia* Clade and *Phanera* Clade, and displayed the strongest support. In the *Phanera* Clade, all species of *Phanera* species form a monophyletic group in the phylogenetic tree and receive good support, forming a sister relationship with *Cheniella* species. Among the species within *Phanera*, *P. erythropoda*, *P. vahlii*, *P. aureifolia*, and *P. bidentata* were clustered to form a closely related sister group. Meanwhile, *P. japonica* and *P. cercidifolia* had the closest genetic relationship with high support rates (PP = 1.00, BS = 100). These species were sister taxa to *P. apertilobata*, but the position of *P. apertilobata* within the phylogenetic tree was only moderately supported (PP = 1.00, BS = 72). *P. championii* and *P. venustula* clustered together as one clade, while *P. yunnanensis* formed a separate clade that was sister to this branch, with high support for this relationship. It was worth noting that *P. saigonensis* had formed a distinct lineage, which was the sisters of other species of *Phanera*.

## 4. Discussion

### 4.1. Composition and Structural Characteristics of Chloroplast Genome

Land plants exhibit a high degree of conservation in the structure and genetics of their cp genomes, despite significant variations in their sizes, independently of the nuclear genome size [52,53]. The cp genomes of the nine *Phanera* species assembled in this study are comparable to those of other *Phanera* species, with sizes varying between 156,240 and 168,155 bp, positioning them among the larger cp genomes found in land plants [52]. Structurally, these cp genomes were similar to that observed in most green plants, featuring a typical circular quadripartite structure comprised of the LSC (86,829–89,725 bp), SSC (11,035–19,010 bp), and a pair of IR regions (25,647–34,934 bp). These cp genomes share a similar gene composition, with a total of 130–135 annotated genes, indicating that both their cp genome structures and gene compositions are conserved. The overall GC content remains consistent across species (35.9–36.3%), with the highest content observed in the IR regions (39.3–42.0%), which may accelerate mutation rates, including single-base substitutions, deletions, and duplications [54]. Despite the high structural conservation of the cp genome, variations in the boundaries of the IR regions are commonly observed during cp genome evolution. These changes, including contractions and expansions, can lead to structural variations in genes [55,56]. Our analysis of these cp genomes revealed that the IR length in *P. bidentata* is longer than in other species (ca. 7 kb), accompanied by the observation of multiple copies of the *ycf1*, *rps15*, and *ndhH* genes. Changes in the size of the IR region may be related to the alteration in the number of boundary genes, a phenomenon observed in numerous plant species [57,58]. Moreover, the SSC region in *P. bidentata* is approximately 6700 bp shorter than in other species, leading us to speculate that this difference arises from the expansion of the IR boundary.

The usage bias of codons reflects the origin, mutation patterns, and evolution of species or genes, showing variations across the genomes of diverse organisms [37,39]. In this study, the nine cp genomes of *Phanera* exhibited a higher frequency of usage (RSCU > 1) for 29 codons, predominantly terminating in A/U, with the codon encoding isoleucine occupying the top spot. Meanwhile, 30 codons were found to be less frequently used (RSCU < 1), largely ending in C/G, with the codon for cysteine being the lowest position.

### 4.2. Phylogenetic Relationships

The ML and Bayesian trees were constructed from the cp genomes of nine *Phanera* species sequenced in this study, and 27 related species performed similar topological structures. All Cercidoideae species were divided into two subtribes: Cercidinae and Bauhiniinae. At the base of the Cercidoideae subfamily sat the Cercidinae, embracing species from *Cercis*, *Adenolobus*, and *Grinffonia*. Subtribe Bauhiniinae further divided into two strongly supported lineages: *Bauhinia* Clade and *Phanera* Clade. The topological structure concurred with previous research findings [3,25]. Within the *Phanera* Clade, all *Phanera* species in this study formed a well-supported monophyletic group, suggesting that the genus *Phanera* may be monophyletic. The genus most closely related to *Phanera* was *Cheniella*, which was reinstated as a genus-level taxon from Clark’s taxonomic revisions [24]. In this study, both *Phanera* and *Cheniella* were resolved as monophyletic sister groups, lending molecular support to the uniqueness of *Cheniella*. Among the species within *Phanera*, *P. saigonensis* formed a distinct lineage, sister to other *Phanera* species. This species is distributed across Cambodia, Laos, Thailand, and Vietnam. The subsequent internal taxa were *P. yunnanensis*, *P. championii*, and *P. venustula* from southern China. The phylogenetic analysis indicated that *P. apertilobata*, distributed in southeastern China, had a phylogenetically close relationship with the Malaysian species *P. bidentata*, followed by *P. aureifolia* from Thailand. They then clustered with *P. aurea* from southwestern China and *P. vahlii* from India. Overall, the phylogenetic positions of these species did not exhibit a strong correlation with geographical distribution. However, there were still some indications that the results hinted at a possible trend of this group’s expansion from Southeast Asia towards South Asia and that South China is a rapid speciation center for *Phanera*. Notably, upon broadening the sampling range of *Phanera*, the species, including *P. apertilobata*, *P. bidentata*, *P. aureifolia*, *P. vahlii*, and *P. erythropoda*, formed a highly supported group. This finding was inconsistent with previous studies, likely due to sampling limitations in those earlier investigations [24,25]. In addition, our analysis revealed that *P. japonica* was more closely related to *P. cercidifolia* than to *P. macrostachya*. *P. apertilobata* received only moderate support in this study, potentially suggesting the need for further research to consolidate its position within the phylogenetic tree.

### 4.3. Development of DNA Markers

Effective hypervariable sequences, vital for species identification and phylogenetic analysis, can be screened out based on cp genome comparisons [59,60]. Employing the mVISTA (https://genome.lbl.gov/vista/mvista/submit.shtml, accessed on 4 October 2024) and DnaSP v5.10.01 software, we identified 10 promising potential variable markers: *rps12*-*clpP*, *clpP*-*psbB*, *trnI-CAU*-*ycf2*, *rps16*-*trnQ-UUG*, *rpoC1*-*rpoB*, *clpP*, *rps18*, *rps16*, *rps15*, and *rps3*. These markers, characterized by high sequence variability, offer a foundation for further phylogenetic research and species identification of *Phanera*.

In addition, the cp genomes of plants are rich in SSRs and dispersed repeats, which have been instrumental in species distinction and genetic diversity analysis of both plant and animal species, owing to their attributes such as high polymorphism, stability, accessibility, and codominant inheritance [20,61,62]. Among the nine cp genomes of *Phanera*, a total of 82–126 SSRs were identified, with a concentration in LSC (accounting for 67.01–81.00% of all regions within the cp genome), primarily composed of polyA and polyT repeats. And mononucleotide SSRs dominated the landscape, accounting for the majority (63.53–64.29%) of all identified SSRs. The prevalence of short repeats as the main repeat type may have indicated the specificity or the differing selective pressures acting upon this species, indicating a relatively diverged evolutionary state for *Phanera*. Additionally, 5448 dispersed repeat sequences, including forward, reverse, palindromic, and complementary repeats, were uncovered in these species, with forward repeats being prominent. These repeats were similar to other Leguminosae cp genomes, providing more available dispersed repeats [63,64]. The SSRs and dispersed repeats identified in this study hold potential as markers for assessing the genetic diversity within *Phanera*.

## 5. Conclusions

In this study, the complete cp genomes of nine *Phanera* species were sequenced and analyzed. The results of assembly and annotation revealed minimal variations in gene order and structure compared to closely related genera, albeit with contractions and expansions observed at the boundaries of SSC and IR regions. The codon usage bias analysis of these species found that the codon usage pattern of these cp genomes favored A/T-ending codons. Further examination of repeat sequences identified 82–126 SSRs and 5448 dispersed repeats, while the discovery of 10 potential variable markers within hypervariable regions could be used as genetic diversity markers. The phylogenetic tree constructed based on the complete cp genomes clarified the evolutionary relationship of *Phanera* and ascertained their phylogenetic positions.

## Figures and Tables

**Table 1:** Taxonomic revision of *Phanera* genus in previous studies.

Loureiro et al., 1790 [4]	Wunderlin et al., 1987 [19]	Lewis and Forest, 2005 [5]	Sinou et al., 2009 [21]	Wunderlin, 2010 [22,23]	Clark et al., 2017 [24]	Sinou et al., 2020 [3]
***Phanera* Lour. (1)**	***Bauhinia*, subg. *Phanera*, sect. *Schnella* (Raddi) Benth. (8) and sect. *Caulotretus* DC. (c. 31)**	***Phanera* (c. 120–130)**	***Phanera* (“American *Phanera* clade”)**	*Schnella* Raddi (c. 40)	*Schnella* (c. 40)	*Schnella* (45)
	***Bauhinia*, subg. *Phanera*, sect. *Phanera* (Lour.) Wunderlin et al., subsect. *Fulvae* (de Wit) Wunderlin et al., ser. *Corymbosae* (de Wit) Wunderlin et al. (c. 6)**		***Phanera* (c. 120–130)**	***Phanera* (c. 90–100)**	*Cheniella* R.Clark and Mackinder (10)	*Cheniella* (10)
	***Bauhinia* subg. *Phanera* (minus sections *Lasiobema*, *Lysiphyllum,* and *Tylosema*) (c. 122)**				***Phanera* (c. 90)**	***Phanera* (74)**
	***Bauhinia*, subg. *Phanera*, sect. *Lasiobema* (Korth.) Benth. (c. 15)**	*Lasiobema* (Korth.) Miq. (c. 15–20)	*Lasiobema* (c. 15–20) (“Asian *Phanera* clade”)			
	***Bauhinia*, subg. *Phanera* (Lour.) Wunderlin et al., sect. *Lysiphyllum* Benth., subsect. *Tournaya* (A.Schmitz) Wunderlin (3)**	*Gigasiphon* Drake (4–5)	*Gigasiphon* (4–5)	*Gigasiphon* (5)	*Gigasiphon* (5)	*Tournaya* A.Schmitz (1)
	***Bauhinia*, subg. *Phanera*, sect. *Tylosema* Schweinf. (4)**	*Tylosema* (Schweinf.) Torre and Hillc. (4)	*Tylosema* (4)	*Tylosema* (4)	*Tylosema* (4)	*Tylosema* (5)
	***Bauhinia*, subg. *Phanera*, sect. *Lysiphyllum* Benth. (9)**	*Lysiphyllum* (Benth.) de Wit (c. 8)	*Lysiphyllum* (c. 8)	*Lysiphyllum* (9)	*Lysiphyllum* (9)	*Lysiphyllum* (8)

This table is an extension of the table presented in Sinou et al., 2020 [3]. The numbers in parentheses represent the species count for this taxonomic group. The genus *Phanera* is highlighted in bold. The red text indicates that the taxon is involved in the revision, while the black text represents taxa that are not involved.

## Data Availability

The chloroplast genome sequences supporting this study have been uploaded to GenBank (National Center for Biotechnology Information) with the accession number PQ433121-PQ433129. The BioProject ID is PRJNA1169201, with BioSample identifiers as SAMN44066747-SAMN44066755. And the corresponding SRA numbers are SRR30895495-SRR30895503.

## Supplementary Materials

The following supporting information can be downloaded at https://www.mdpi.com/article/10.3390/genes15111456/s1, Table S1: General information of chloroplast genomes of *Phanera* species.; Table S2: Genes present in the chloroplast genomes of nine *Phanera* species; Table S3: Codons in cp genome of *P. erythropoda*; Table S4: Codons in cp genome of *P. vahlii*; Table S5: Codons in cp genome of *P. aureifolia*; Table S6: Codons in cp genome of *P. bidentata*; Table S7: Codons in cp genome of *P. japonica*; Table S8: Codons in cp genome of *P. saigonensis*; Table S9: Codons in cp genome of *P. yunnanensis*; Table S10: Codons in cp genome of *P. apertilobata*; Table S11: Codons in cp genome of *P. championii*; Figure S1: Dispersed repeat sequence statistics data in nine *Phanera* species; Figure S2: Dispersed repeat sequence distribution in nine *Phanera* species; Figure S3: SSRs distribution in nine *Phanera* species.
