# Supplementary material for: Complete Chloroplast Genomes and Phylogenetic Analysis of Woody Climbing Genus Phanera (Leguminosae)
Source: Genes (Basel). 2024 Nov 12;15(11):1456. doi: 10.3390/genes15111456 (PMC11593341; doi:10.3390/genes15111456)
Supplement: Supplementary file 1 [file genes-15-01456-s001.zip › Caption of Supplementary Materials.docx]

**Table S1:** General information of chloroplast genomes of *Phanera* species.;

**Table S2:** Genes present in the chloroplast genomes of nine *Phanera* species;

**Table S3:** Codons in cp genome of *P. erythropoda*;

**Table S4:** Codons in cp genome of *P. vahlii*;

**Table S5:** Codons in cp genome of *P. aureifolia*;

**Table S6:** Codons in cp genome of *P. bidentata*;

**Table S7:** Codons in cp genome of *P. japonica*;

**Table S8:** Codons in cp genome of *P. saigonensis*;

**Table S9:** Codons in cp genome of *P. yunnanensis*;

**Table S10:** Codons in cp genome of *P. apertilobata*;

**Table S11:** Codons in cp genome of *P. championii*;

**Figure S1:** Dispersed repeat sequence statistics data in nine *Phanera* species;

**Figure S2:** Dispersed repeat sequence distribution in nine *Phanera* species;

**Figure S3:** SSRs distribution in nine *Phanera* species.
