# Supplementary figures and images for: Complete Chloroplast Genomes and Phylogenetic Analysis of Woody Climbing Genus Phanera (Leguminosae)
Source: Genes (Basel). 2024 Nov 12;15(11):1456. doi: 10.3390/genes15111456 (PMC11593341; doi:10.3390/genes15111456)

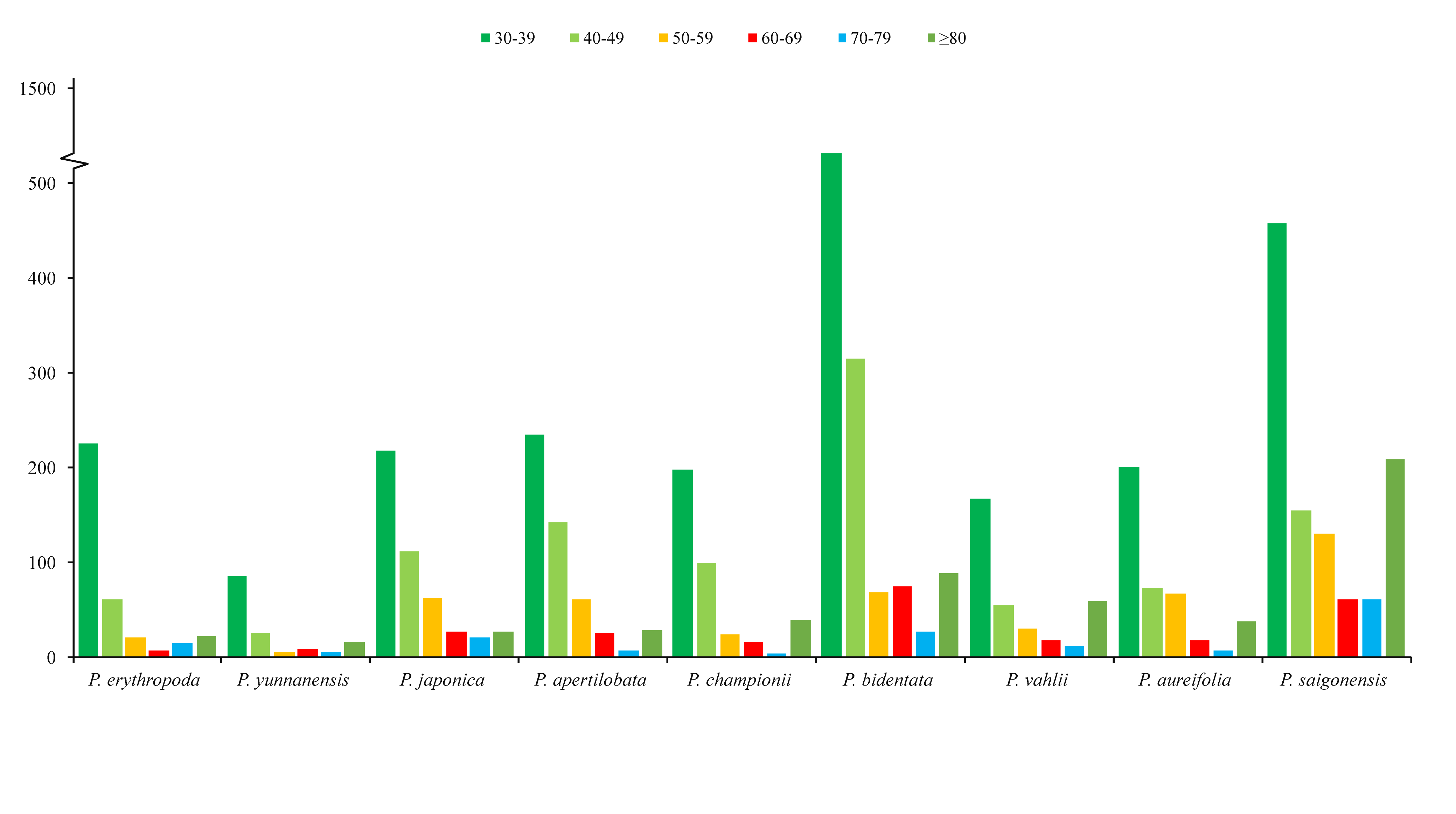

Supplement: Supplementary file 1 [file genes-15-01456-s001.zip › Figure S1.tif]

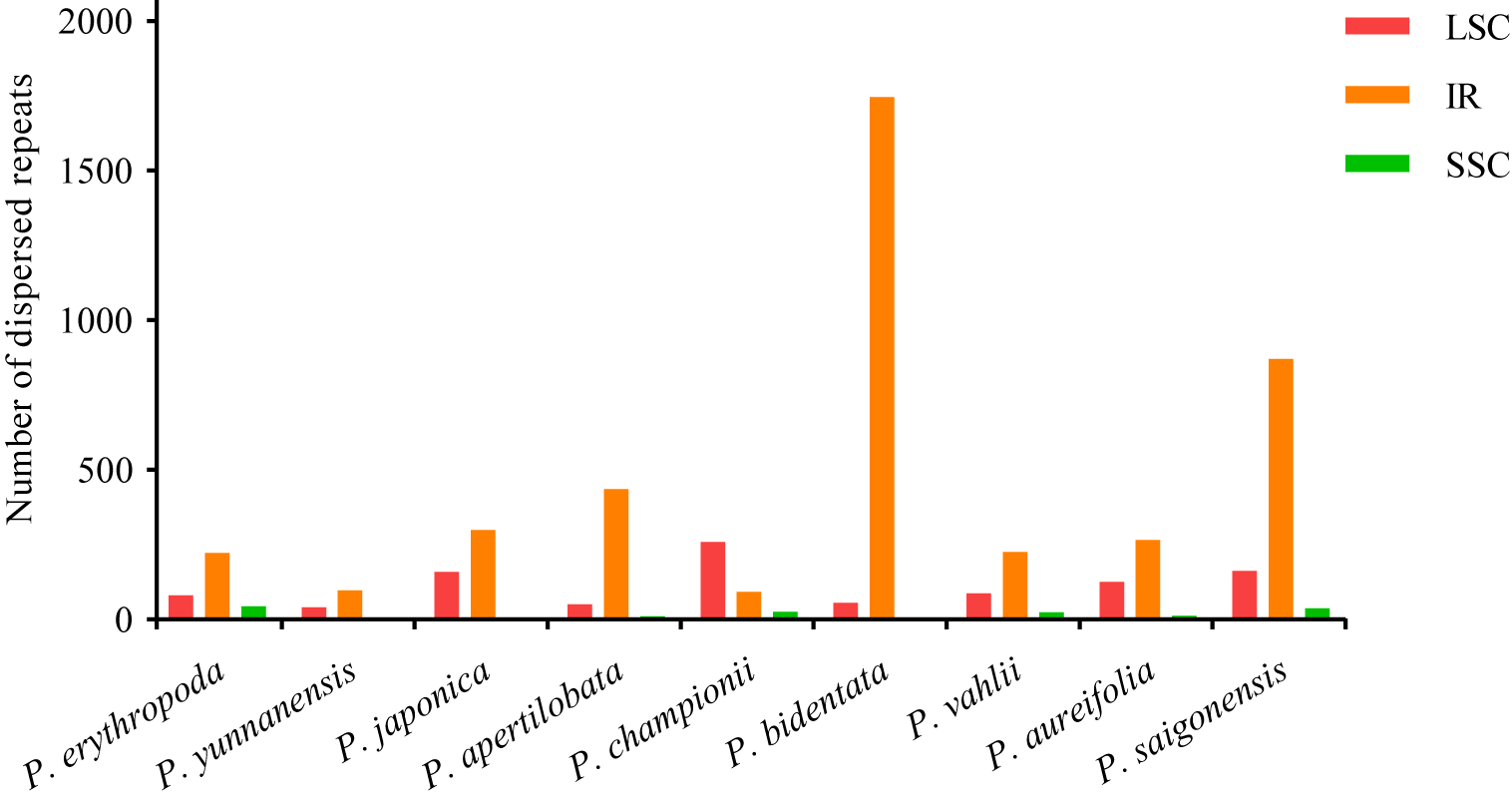

Supplement: Supplementary file 1 [file genes-15-01456-s001.zip › Figure S2.tif]

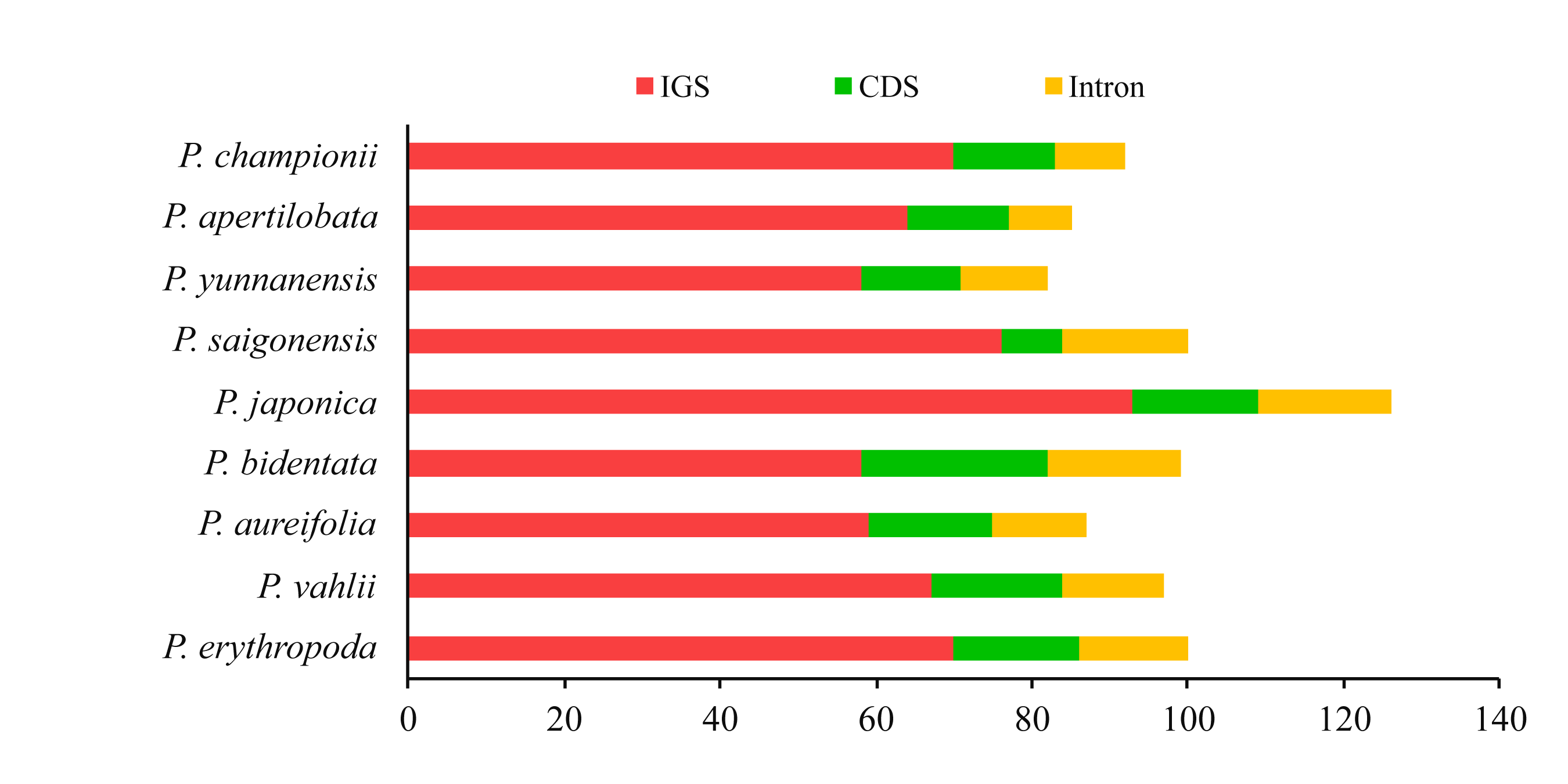

Supplement: Supplementary file 1 [file genes-15-01456-s001.zip › Figure S3.tif]
